# Parent-of-origin effects on genome-wide DNA methylation in the Cape honey bee (*Apis mellifera capensis*) may be confounded by allele-specific methylation

**DOI:** 10.1186/s12864-016-2506-8

**Published:** 2016-03-12

**Authors:** Emily J. Remnant, Alyson Ashe, Paul E. Young, Gabriele Buchmann, Madeleine Beekman, Michael H. Allsopp, Catherine M. Suter, Robert A. Drewell, Benjamin P. Oldroyd

**Affiliations:** Behavior and Genetics of Social Insects Laboratory, School of Life and Environmental Sciences A12, University of Sydney, Room 248, Macleay Building (A12), Sydney, NSW 2006 Australia; School of Life and Environmental Sciences, University of Sydney, Sydney, NSW 2006 Australia; Victor Chang Cardiac Research Institute, Lowy Packer Building, 405 Liverpool Street, Darlinghurst, NSW 2010 Australia; University of New South Wales, Kensington, NSW 2033 Australia; Honey Bee Research Section, ARC-Plant Protection Research Institute, Private Bag X5017, Stellenbosch, South Africa; Biology Department, Clark University, 950 Main Street, Worcester, MA 01610 USA

**Keywords:** *Apis mellifera capensis*, Imprinting, Parent-of-origin effects, Allele-specific methylation, Thelytokous parthenogenesis

## Abstract

**Background:**

Intersexual genomic conflict sometimes leads to unequal expression of paternal and maternal alleles in offspring, resulting in parent-of-origin effects. In honey bees reciprocal crosses can show strong parent-of-origin effects, supporting theoretical predictions that genomic imprinting occurs in this species. Mechanisms behind imprinting in honey bees are unclear but differential DNA methylation in eggs and sperm suggests that DNA methylation could be involved. Nonetheless, because DNA methylation is multifunctional, it is difficult to separate imprinting from other roles of methylation. Here we use a novel approach to investigate parent-of-origin DNA methylation in honey bees. In the subspecies *Apis mellifera capensis,* reproduction of females occurs either sexually by fertilization of eggs with sperm, or via thelytokous parthenogenesis, producing female embryos derived from two maternal genomes.

**Results:**

We compared genome-wide methylation patterns of sexually-produced, diploid embryos laid by a queen, with parthenogenetically-produced diploid embryos laid by her daughters. Thelytokous embryos inheriting two maternal genomes had fewer hypermethylated genes compared to fertilized embryos, supporting the prediction that fertilized embryos have increased methylation due to inheritance of a paternal genome. However, bisulfite PCR and sequencing of a differentially methylated gene, *Stan* (GB18207) showed strong allele-specific methylation that was maintained in both fertilized and thelytokous embryos. For this gene, methylation was associated with haplotype, not parent of origin.

**Conclusions:**

The results of our study are consistent with predictions from the kin theory of genomic imprinting. However, our demonstration of allele-specific methylation based on sequence shows that genome-wide differential methylation studies can potentially confound imprinting and allele-specific methylation. It further suggests that methylation patterns are heritable or that specific sequence motifs are targets for methylation in some genes.

**Electronic supplementary material:**

The online version of this article (doi:10.1186/s12864-016-2506-8) contains supplementary material, which is available to authorized users.

## Background

Sexual reproduction is generally characterized by the equal transmission of nuclear genomes from mothers and fathers to offspring, and by equal expression of maternal and paternal alleles in offspring [1, 2]. However violations of Mendelian inheritance can arise when the genetic interests of the mother and father diverge [2, 3]. Males benefit if their offspring can secure more resources from the offspring’s mother, whereas females normally benefit if their offspring are provisioned equally regardless of paternity. These sexual conflicts can lead to parent-of-origin effects, arising from genomic imprinting, where offspring phenotype is altered depending on whether an allele is inherited from a mother or a father [4]. Imprints are laid down during gametogenesis, during an epigenetic reprogramming event that results in the non-equivalence of paternal and maternal genomes [5]. For example, mice embryos derived from two maternal genomes or two paternal genomes via pronuclear transfers are non-viable due to parent-specific imprinting [6, 7].

Parent-specific epigenetic modifications that give rise to genomic imprinting often occur via DNA methylation, which involves the addition of a methyl group to cytosine residues of DNA [8]. In addition to its role in imprinting, DNA methylation varies between cell types due to cellular differentiation [9], and may also vary according to genotype due to allele-specific methylation [10]. DNA methylation can alter the expression and regulation of genes [11]. When methylation occurs in promoter regions, it results in suppression of gene expression. When methylation occurs in the exons and introns of gene bodies, it increases or maintains gene expression and may mediate alternate splicing [12–14]. Gene body methylation is conserved among plants, invertebrates and mammals, and may therefore represent an ancestral function of DNA methylation [15, 16]. Not all invertebrates have DNA methylation, but if it is present it is generally restricted to gene bodies and affects only a small percentage of cytosines genome-wide [17].

Colonies of Hymenopteran insects (bees, wasps and ants) provide a situation that is ripe with potential for intra-genomic conflict and thus for imprinting [18–20]. Hymenoptera are haplo-diploid. Females arise from fertilized eggs and are diploid, while males derive from unfertilized eggs by arrhenotokous parthenogenesis and are haploid. Workers almost never lay eggs when a queen is present but are physiologically able to produce haploid eggs that give rise to males. This ability allows workers of some species to occasionally ‘cheat’ and lay haploid eggs that produce viable males [21, 22]. In many eusocial hymenopterans the queens are polyandrous [23]. Because females mate with multiple males, any male who can epigenetically modify his female offspring so that they are more likely to become reproductively active will be favored by selection, as his offspring will outcompete those of males that do not do so [18, 20].

**Fig. 1:**
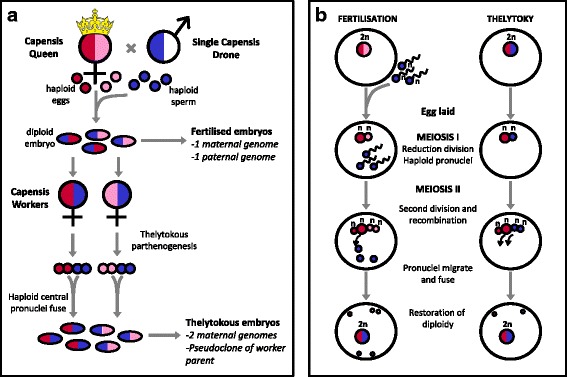
Design of the experiment. **a** A Capensis queen was inseminated with sperm from a single Capensis drone, to produce fertilized embryos. A subset of embryos were collected to produce the fertilized embryo methylome, while the remaining embryos were left to emerge to adult workers. When the queen was removed, the daughter workers began to produce diploid eggs thelytokously and these embryos were used to produce the thelytokous embryo methylome. **b** Early meiotic divisions in fertilisation and thelytoky follow the same process. The first meiotic division occurs after oviposition in a newly laid egg. If the egg is fertilized there will be 3–7 spermatozoa, one of which becomes the male pronucleus. During thelytokous reproduction, the egg is unfertilized and two maternal pronuclei fuse to form a diploid nucleus [31]

Unlike mammals where parthenogenesis generates inviable offspring due to imprinting [24–26], some Hymenopterans can produce diploid female offspring from unfertilized eggs by thelytokous parthenogenesis [27]. Thelytoky, the asexual production of female offspring, is rare in honey bees generally but ubiquitous in workers of the subspecies *Apis mellifera capensis* (hereafter Capensis) [28, 29]. In Capensis, mated queens reproduce sexually, identically to all other honey bee subspecies, by the union of egg and sperm nuclei within a newly-laid egg. In contrast to other honey bee species, Capensis workers lay diploid eggs that develop into females ([30, 31]; Fig. 1 ), by the fusion of two maternally-derived pronuclei, as if one pronucleus acted as a sperm, again within a newly-laid egg [31]. Embryogenesis otherwise occurs normally as it would in a fertilized embryo, resulting in the production of diploid females.

Thelytokous parthenogenesis as described above generates embryos that are ‘pseudoclones’ of their worker parent. That is, thelytokous offspring receive one copy of the two grandparental genomes and are largely identical to their thelytokous parent, unless recombination during meiosis has resulted in loss of heterozygosity [32]. While thelytoky enables workers to genetically re-incarnate themselves, it is currently unknown how the epigenetic component of the genome is inherited following thelytokous meiosis. Sexually-produced embryos laid by a queen inherit both a maternally- and a paternally-derived set of chromosomes, whereas thelytokous eggs contain pronuclei that have undergone gametogenesis within the ovaries of female workers. Thus thelytokous embryos begin life with two maternally-inherited pronuclei, (Fig. 1) and may show different patterns of methylation to sexually-produced embryos. Therefore Capensis provides the ideal system in which to examine parent-of-origin effects, as it naturally generates diploid bi-parental and diploid uni-parental embryos.

As yet we have only indirect evidence for genomic imprinting and parent-of-origin effect in social insects in general, and honey bees in particular. However three observations suggest the presence of imprinting in the honey bee. First, methylation patterns differ significantly between unfertilized eggs and sperm, suggesting that queens and drones differentially methylate their gametes [33]. Second, reciprocal crosses between honey bee subspecies show substantial parent-of-origin effects for reproductive traits, again suggestive of paternal imprinting [34–36]. Finally, in reciprocal crosses of Africanised and European honey bees, 46 transcripts showed significant parent-of-origin expression effects [37]. A second study using Africanised and European honey reciprocal crosses determined that hybrid workers showed paternally biased phenotypes and expression patterns, particularly relating to reproduction, suggesting that in honey bees worker reproduction is driven by patrigenes [38].

In addition to its potential role in genomic imprinting, DNA methylation has additional functions in Hymenoptera [39–41]. In honey bees it is involved in larval development [42], and differentiation into queen and worker castes [43]. Methylation patterns differ between the brains of honey bee queens and workers [44], as well as between adult workers during behavioural maturation [45]. The majority of genomic methylation is associated with constitutively-expressed, highly-conserved genes [12, 46–49] and, approximately half of all genes are methylated [33]. Methylation may provide a mechanism by which gene dosage is regulated between the haploid and diploid castes [50]. How methylation is regulated, and the mechanisms by which DNA methylation is directed to particular sites and genes is unknown.

Here we examine the methylation patterns of fertilized, sexually produced, bi-parental embryos laid by Capensis queens with asexually produced, parthenogenetic, uni-parental embryos produced by their thelytokous daughter workers (Fig. 1). We determine whether thelytokous embryos with two maternal genomes have different patterns of methylation to sexually-produced embryos with a maternal and paternal genome. To control for genotype, experimental queens were artificially inseminated, each with sperm from a single drone. Therefore, the worker progeny were genetically homogenous, each worker carrying one allele from the inseminating male, and one of the queen’s two alleles. At a cohort level, the workers carried just three alleles, two that were maternally derived, and one that was paternally derived. On average, the worker-laid embryos were genetically identical to those of the queen-derived embryos, and differed only in epigenetic modifications. The presence of three alleles in common through two generations enabled us to follow the methylation patterns of each allele in both fertilized and thelytokous embryos.

If paternal imprinting exists, we would predict significantly more methylated sites in sexually-produced embryos due to methylation present in the paternally-inherited sperm pronucleus. In thelytokous embryos, we expect fewer methylated sites overall. Conversely, for maternally imprinted alleles we would predict significantly higher levels of methylation at specific maternally imprinted sites in thelytokous embryos, due to a double dosage of two maternally imprinted genomes. An absence of differential methylation between thelytokous and fertilized diploid embryos would be strong evidence that imprinting does not occur in the honey bee. Evidence of allele-specific methylation would show that epigenetic modifications are heritable in the honey bee or that they are invariably established according to cis-mediated genotype, but could also confound our ability to detect imprinting.

## Methods

### Sample collection, DNA extraction and sequencing

We produced three colonies (1–3) by insemination of three virgin Capensis queens, each with the semen of a single Capensis drone. The parents were sourced in Stellenbosch, South Africa. The inseminated queens were introduced into separate queenless host colonies. After the introduced queens began laying we collected between 300 and 1200 fertilized embryos from each colony (0–70 h old, hereafter ‘fertilized embryos’) into 99 % ethanol.

Once all host workers had been replaced by worker-offspring of the inseminated queens (about two months), we removed the queens from the colonies. The now queenless Capensis workers commenced laying thelytokous eggs approximately 1 week after the removal of the queen. These Capensis daughter workers produced diploid eggs via thelytokous parthenogenesis. We collected between 300 and 900 worker-laid embryos from each colony (0–70 h old, hereafter ‘thelytokous embryos’), again into ethanol.

We used microsatellite analysis to confirm that individual embryos produced by workers from this colony were all thelytokous diploid females and not arrhenotokous haploid males [29]. We also confirmed that DNA from the fertilized and thelytokous embryos contained the same microsatellite alleles at five unlinked loci, and that there were no more than three alleles at each microsatellite confirming two alleles were derived from the queen and one allele from the sperm from the single drone [29]. This proves that our colonies had not been invaded by reproductive parasites, as often happens in queenless *A. m. capensis* colonies [51, 52].

Our design resulted in three independent sets of paired embryos (‘fertilised’ and ‘thelytokous’) that were diploid and genetically (but not necessarily epigenetically) equivalent (Fig. 1a). Embryos were collected over similar time frames, so if methylation patterns change during embryonic development, these changes would be equivalent in both cohorts.

Colony 1 generated sufficient fertilized (1280) and thelytokous (870) embryos for whole genome bisulfite sequencing. To extract DNA we removed the ethanol, rinsed the embryos in water, and resuspended them in G2 buffer (Qiagen). Embryos were lysed in a tissue lyser and treated with 2 mg/ml RNAse at 37 °C for 30 min, Proteinase K at 56 °C for two hours, and DNA purified using 20G genomic tips (Qiagen). We quantified the amount of DNA in the extracts using the Qubit Flurometer (Life Technologies). The final yield for fertilized and thelytokous embryo samples was 8.3 and 2.5 μg, respectively. We added lambda bacteriophage DNA (0.1 % (w/w), Promega) to each sample as a spike-in control to evaluate the efficacy of the bisulfite conversion [53]. Bisulfite conversion, library construction and sequencing were performed at the Beijing Genomics Institute as described in Drewell et al. [33].

Embryos from colonies 2 and 3 were reserved for bisulfite PCR validation of candidate genes.

### Whole genome bisulfite sequence mapping

We used BSMAP v2.74 to determine methylation state of cytosine residues from the whole genome sequencing [54]. Bisulfite reads were aligned to the *A. mellifera* genome assembly 2.0 [55] as described in Drewell et al. [33].

*A. m. capensis* is a different subspecies to the reference genome - *A. m. ligustica* [55, 56]. If there is significant polymorphism between the Capensis and reference genomes then the proportion of bisulfite reads that could be successfully aligned to the reference genome would be reduced. To assess the extent of variation between the Capensis and reference genomes, we obtained *A. m. capensis* genome sequence data from Wallberg *et al,* [57] and generated a ‘Capensis2.0’ reference genome. Briefly, we aligned the *A. m capensis* reads [57] to the *A. mellifera* genome assembly 2.0 using BWA v0.5.9 [58]. The alignment of the 75 bp Capensis reads resulted in 16.8-fold coverage of the reference genome. We utilized the alignment to determine SNPs and attempted to generate an alternate ‘Capensis2.0’ reference. File conversions, SNP calling and creation of an alternate reference was performed with SAMtools v0.1.16 [59] and GATK v3.3 [60]. We repeated BSMAP bisulfite mapping to the generated Capensis2.0 reference. Compared to the original mapping results, fold coverage and methylation calls (CG/CHG/CHH) were not significantly improved (Additional file 1: Table S1). We therefore continued analysis based on the alignment to the *A. mellifera* Assembly 2.0, so that our data can be directly compared with existing methylomes [33, 42, 44].

We determined the bisulfite conversion rate in our DNA samples by calculating the C-to-T conversion rate in the lambda spike-in control. Using BSMAP we aligned reads to the bacterophage lambda genome (Genbank J02459.1). Based on the number of converted cytosines to total lambda cytosine coverage, we estimated the bisulfite conversion rate as 0.9970 for the fertilized embryo sample, and 0.9965 for the thelytokous embryo sample.

### Methylation analysis

The number of methylated cytosines in each sample was calculated as in Drewell et al. [33]. At each cytosine site, we compared the number of bisulfite-converted reads (unmethylated) and the number of non-converted reads (methylated). We performed a binomial test to determine significant methylation, with a probability of one minus the lambda phage DNA conversion rate, correcting for multiple comparisons [61].

To quantitate differential methylation between the two samples, the output from BSMAP was analysed using the R package methylKit [62]. To compare the frequency of methylated cytosines at CpG sites, we first excluded sites with less than 5 fold coverage. Sites containing a methylation difference of 25 % or greater, with a *P* value of 0.05 or less were deemed to be differentially methylated, and annotated against the official gene set from *A. mellifera* assembly 2.0.

### Bisulfite PCR validation

Bisulfite PCR was conducted on samples of fertilized and thelytokous eggs from the two remaining colonies, 2 and 3. DNA was extracted from 300 embryos per sample, using the DNeasy blood and tissue kit (Qiagen). Two independent bisulfite conversion reactions were performed per sample (Zymo EZ DNA Methylation-Direct kit). Bisulfite PCR assays were designed for five candidate genes, *Stan* (GB18207), *Stoned B* (GB17165), *Sap30* (GB18386), *Syd* (GB15356) and *Pcl* (GB16657; Table 3). Nested primers were selected spanning a region containing as many differentially methylated sites as possible within each candidate gene and PCR products were amplified (Additional file 1: Table S2). Previous studies have noted the difficulties of amplifying regions greater than 500–600 bp in length from bisulfite converted DNA [63, 64]. Using the two-step nested PCR and KAPA 2G Robust DNA polymerase (KAPA biosystems), we were able to amplify 793 bp (*Stan*), 650 bp (*Stoned B*), 1022 bp (*Sap30*), 1224 bp (*Syd*) and 1040 and 1375 bp (*Pcl*), fragments from the candidate genes (Additional file 1: Table S2). For three genes (*Stan, Stoned B* and *Sap30*), PCR products from each independent bisulfite reaction, for each sample were separately cloned into the TOPO vector (Invitrogen) and a minimum of 20 individual amplicons were sequenced for each sample (Macrogen, Seoul). Fragments amplified from the other two genes (*Syd* and *Pcl*) could not be cloned or did not generate sufficient clones for analysis after repeated attempts.

## Results

### CpG methylation frequency in the Capensis genome

On average we obtained 8.6-fold and 10.1-fold genome coverage for fertilized and thelytokous embryo samples, respectively, achieving a similar level of coverage to previous honey bee methylation studies [33, 44, 45]. Genome-wide, the proportion of total CG sites covered by 2 to 30 reads was 79.4 % for fertilized embryos, and 80.3 % for thelytokous embryos (Table 1). There were 114,156 methylated cytosines in the CG context in fertilized embryos and 99,923 in thelytokous embryos. The proportion of CG sites in the genome that were methylated was 0.72 % for fertilized embryos and 0.62 % for thelytokous embryos, consistent with previous studies of the honey bee methylome [33, 44]. Taking into account the total genome-wide number of CG sites covered by 2–30 reads in both samples, the number of methylated CG sites was significantly greater in fertilized embryos than in thelytokous embryos (*χ*_1_^2^ = 1133.0, *p* <0.001).

**Table 1:** Coverage and context of cytosine methylation in fertilized and thelytokous embryo samples

		Cytosine context (number of sites with 2–30 fold coverage)			
		CG	CHG	CHH	All cytosines
Genome (Amel2.0)	Total	20,060,266	8,673,586	45,077,073	73,810,925
Fertilized embryos	Total covered	15,923,958	6,805,662	31,027,094	53,756,741
	% of genome covered	79.4	78.5	68.8	72.8
	Total methylated	**114,156**	3,131	41,607	158,894
	% methylation	0.72	0.05	0.13	0.30
Thelytokous embryos	Total covered	16,116,870	6,758,019	31,180,274	1,523
	% of genome covered	80.3	77.9	69.2	73.2
	Total methylated	**99,923**	886	12,551	113,361
	% methylation	0.62	0.01	0.04	0.21
Fertilized vs Thelytokous	Methylated in both	**74,798**	86	475	75,060
	Unique to Fertilized	21,202	2,149	24,526	47,880
	Unique to Thelytokous	16,004	682	9,699	26,385

**Fig. 2:**
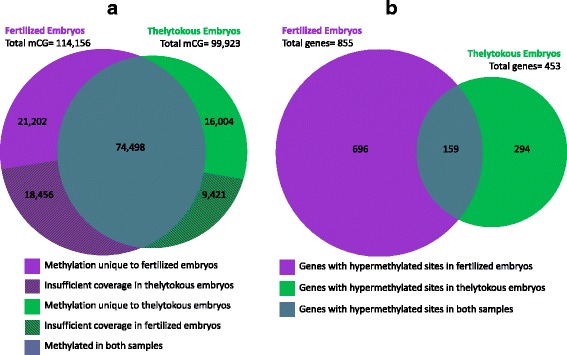
**a** The number of methylated CG sites in fertilized (*purple*) and thelytokous (*green*) embryos. 74,498 sites were methylated in both samples. Fertilized embryos had 21,202 unique methylated CG sites (18,456 additional sites were methylated in fertilized embryos with insufficient coverage to determine methylation in the thelytokous embryo sample). Thelytokous embryos had 16,004 unique methylated CG sites (9,471 additional sites were methylated in thelytokous embryos with insufficient coverage to determine methylation in the fertilized embryo sample). **b** The number of genes containing hypermethylated CG sites in fertilized and thelytokous embryos. Six hundred ninety-six genes contained hypermethylated CG sites in fertilized embryos, while 294 genes contained hypermethylated CG sites in thelytokous embryos. One hundred fifty-nine genes contained some hypermethylated CG sites in fertilized embryos, and some hypermethylated CG sites in thelytokous embryos

Numbers in bold refer to CG methylation presented in Fig. 2a

We define methylated sites as those where the proportion of non-converted bisulfite reads was significantly higher (as determined by a binomial test) than the background bisulfite non-conversion rate (as measured in the lambda control). Ignoring sites that had less than two reads in either sample, 74,498 CG sites were significantly methylated in both samples (Table 1, Fig. 2a). There were 21,202 CG sites methylated in fertilized embryos but not thelytokous embryos, and 16,004 sites methylated in thelytokous embryos but not fertilized embryos (Table 1, Fig. 2a).

**Fig. 3:**
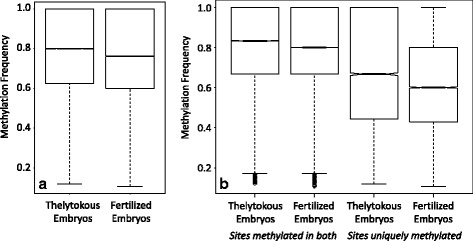
**a** Range of methylation frequencies in methylated CG sites in thelytokous and fertilized embryo methylomes. **b** Range of methylation frequencies in universally methylated CG sites methylated in both thelytokous and fertilized embryos, compared to unique methylated CG sites in fertilized and thelytokous embryos. Universal sites showed higher median methylation frequency compared to uniquely methylated sites

Across methylated CG regions, individual cytosines varied in methylation frequency, ranging from 10 to 100 % methylation (Fig. 3a). The methylated sites that were common to both fertilized and thelytokous embryos had a median methylation frequency of 83 % in thelytokous embryos, and 80 % in fertilized embryos (Fig. 3b). In contrast, the methylated sites that were unique to either fertilized or thelytokous embryos tended to have less methylation. In thelytokous embryos the median methylation frequency of sites showing some methylation was 67 %. In fertilized embryos the median methylation frequency was 60 % (Fig. 3b). This indicates that Capensis embryos share a core set of common CG sites that are highly methylated, regardless of whether the embryos arise sexually or thelytokously. Sites that are methylated only in fertilized or thelytokous embryos tend to have lower methylation frequency (Fig. 3b).

### Non-CpG methylation

We identified over 50,000 cytosine methylation sites in a non-CG context (‘CH’ methylation: cytosines methylated adjacent to A, T or C; Additional file 1: Table S1). Some of these sites were artifacts arising from vector/mammalian contamination that are present in the Amel2.0 reference sequence (769 cytosines; Additional file 1: Table S1). To examine whether the remaining non-CG sites corresponded to actual Capensis CG sites that are absent from the Amel2.0 reference genome (thus artificially appearing as non-CG methylation), we examined the SNPs present from our Capensis2.0 alignment derived from Wallberg et al. [57]. Only 361 (fertilized embryo) and 113 (thelytokous embryo) non-CG sites corresponded to SNPs in the Capensis2.0 reference sequence (Additional file 1: Table S1). It is likely that at least some non-CG methylated sites correspond to SNPs present in our source population that were not present in the Capensis population used by Wallberg et al. [57]. Finally, a small number of false positives are expected to arise from incomplete bisulfite conversion and errors generated by the next generation sequencing process [65]. Despite these caveats we observed a large number of non-CG methylated cytosines in fertilized and thelytokous embroys (28 % of methylated Cs in fertilized embryos, 12 % in thelytokous embryos, Additional file 1: Table S1). Note, however, that very few non-CG methylated sites (561) were present in both samples.

### Differentially methylated cytosines

**Fig. 4:**
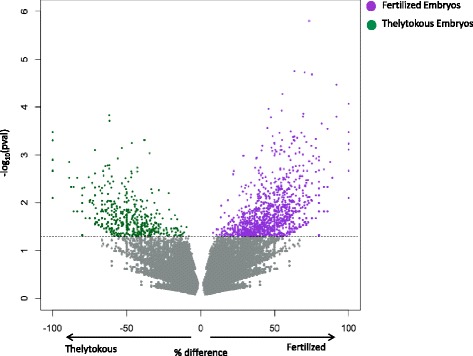
Comparison of differentially methylated CG sites with at least five-fold coverage in fertilized and thelytokous embryos. Percent difference (X axis) is calculated by percent methylation in fertilized embryos minus thelytokous embryos. Non-significant sites are indicated in grey. A total of 2,121 sites had significantly different methylation between the two samples, with 2014 hypermethylated sites having at least 25 % difference in methylation frequency between the two samples. Fertilized embryos had 1412 hypermethylated sites (*purple*) compared to thelytokous embryos with 602 hypermethylated sites (*green*)

Comparison of significantly methylated sites between genomes that have variable coverage may yield false positives due to insufficient coverage in one of the genomes being compared. To avoid biased differential methylation calls based on coverage differences, we determined that the median coverage across methylated CG sites in fertilized and thelytokous embryos was five-fold (Additional file 1: Figure S1), so we restricted our comparison to sites with five or more reads in both samples. Using the R package methylKit [62], we determined the number of significantly differentially methylated CG sites between fertilized and thelytokous embryos. A total of 2,121 sites had significantly different methylation (Fig. 4). 2014 of those sites contained at least a 25 % difference in methylation frequency. If a differentially methylated CG site contained 25 % higher methylation in one sample compared to the other, such sites were considered hypermethylated. Fertilized embryos had significantly more hypermethylated sites (1412) compared to thelytokous embryos (602; *χ*_1_^2^ = 325.8, *P* <0.001, Fig. 4).

### Genes containing differentially methylated sites

**Fig. 5:**
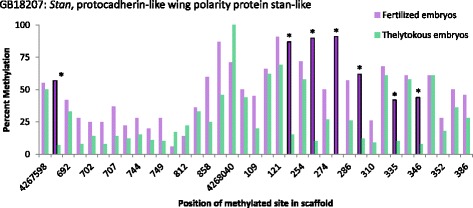
Percent methylation at individual CG sites in *Stan* (GB18207) from whole genome bisulfite sequencing of colony 1. Fertilized embryos (*purple*) show seven significantly hypermethylated CG sites compared to thelytokous embryos (*green*)

We examined the genomic context of differentially methylated sites and found that in both samples over 75 % of sites were located in exons and over 90 % in gene bodies (introns or exons, Table 2). We could therefore assign the majority of differentially methylated sites to annotated genes. As expected from the observation that there are significantly more hypermethylated CG sites in fertilized embryos, there was a significant bias towards hypermethylation of genes in fertilized embryos (*χ*_1_^2^ = 123.6, *p* <0.001, Table 2, Fig. 2b). The top genes containing at least four hypermethylated sites in either fertilized or thelytokous embryos, are shown in Table 3 (for a full list of all genes with hypermethylated sites see Additional file 2 ). Eight genes with four or more hypermethylated sites in fertilized and thelytokous embryos were present among the top 381 differentially methylated genes (DMGs) from our previous comparison of haploid eggs and sperm [33]. In that study, these eight genes all had significantly higher methylation in haploid eggs compared to sperm. The 14^th^ DMG in haploid eggs relative to sperm [33], *Stan* (GB18207) contained 7 hypermethylated sites in fertilized embryos (Table 3, Fig. 5). The top two DMGs in haploid eggs, *Stoned B* (GB17165) and *Sap-30* (GB18386) [33], were both among the most hypermethylated genes in fertilized Capensis embryos with four and five hypermethylated sites, respectively (Table 3).

**Table 2:** Genomic context of hypermethylated CG sites in fertilized and thelytokous embryos

	Fertilised	Thelytokous	Both^a^
Number of hypermethylated sites	1412	603	2015^b^
- Exons *(%)*	1080 *(76.5 %)*	490 *(81.3 %)*	1570 *(78.0 %)*
- Introns *(%)*	197 *(14.0 %)*	63 *(10.4 %)*	260 *(12.9 %)*
- Intergenic *(%)*	135 *(9.5 %)*	50 *(8.3 %)*	185 *(9.1 %)*
Number of genes with hypermethylated sites	855	453	159^c^

^a^Both refers to:

^b^total number of hypermethylated sites in both samples; and

^c^overlap of genes with hypermethylated sites in both samples

**Table 3:** Top differentially methylated genes with 4 or more hypermethylated sites

		Number of hypermethylated sites						
	Gene (GB)	Fertilized embryos	Thelytokous embryos	% diff meth/no. of sites	ID	Description	Region of gene containing diff meth sites (bp)	Rank in haploid egg v sperm [33]
Fertilized embryos	GB19623	10	2	30.0	LOC726254	E3 ubiquitin-protein ligase HECTD1-like	10,393	
	GB18207	7		58.9	LOC551848	Stan, protocadherin-like wing polarity protein stan-like	655	14 (egg)
	GB13657	7		43.3	LOC410109	Ubiquitin carboxyl-terminal hydrolase 34-like	1,038	
	GB12030	7		36.8	Rga	Regulator of gene activity, CCR4-NOT transcription complex subunit 2	1,500	220 (egg)
	GB19502	7	1	27.2	LOC411678	Serine/threonine-protein kinase LATS1-like	1,455	
	GB13625	6		57.6	LOC408938	Sodium/potassium/calcium exchanger-like	75	
	GB12133	6		54.8	LOC726694	Uncharacterized LOC726694	1,992	
	GB11408	6	4	5.9	mask	Multiple ankyrin repeats single KH domain	12,888	
	GB13135	5		65.4	Rho-1	Ras-like GTP-binding protein Rho1	27	
	GB15356	5		54.1	syd	JNK-interacting protein 3	414	
	GB17138	5		53.8	LOC726524	Chromo domain-helicase-DNA-binding protein 7	1,903	
	GB15737	5		53.2	LOC413786	Similar to netrin 1a	192	
	GB17802	5		46.9	hyd	E3 ubiquitin-protein ligase hyd	9,018	
	GB11916	5		45.0	LOC411894	Dynein beta chain, ciliary-like	89	
	GB15553	5		44.8	LOC551066	Female sterile (1) K10 ortholog	2,519	
	GB18689	5		43.9	LOC413663	Myotubularin-related protein 4-like	317	
	GB16783	5	2	18.6	Arp5	Actin-related protein 5	366	
	GB18386	5	2	14.1	Sap30	SAP30-binding protein-like	952	2 (egg)
	GB13213	4		60.2	Eif4g	Eukaryotic translation initiation factor 4 gamma	2735	
	GB15134	4		60.0	LOC413618	Polyadenylation factor subunit 2	3,855	
	GB14328	4		60.0	LOC408308	Coiled-coil-helix-coiled-coil-helix domain-containing protein 2, mitochondrial-like	29	
	GB16657	4		53.8	Pcl	Polycomblike	1,365	
	GB17954	4		53.2	Spt5	Transcription elongation factor SPT5	1,608	183 (egg)
	GB16844	4		48.6	EF1a-F2	Elongation factor 1-alpha F2	680	
	GB14570	4		46.2	LOC551973	Cleavage and polyadenylation specificity factor subunit CG7185 ortholog	3,366	
	GB15773	4	1	45.6	LOC411939	Protein RIC1 homolog	3,465	
	GB18802	4		45.2	LOC409151	Uncharacterized LOC409151	1,307	
	GB17582	4		43.8	LOC411099	Zinc finger protein 84-like	1,739	
	GB17165	4		41.2	stnB	Stoned B	244	1 (egg)
	GB12228	4		41.1	LOC726280	Uncharacterized LOC726280	3,048	
	GB12280	4	1	39.0	spas	Spastin	9,053	
	GB18988	4	1	31.2	CNOT1	CCR4-NOT transcription complex, subunit 1	1,916	
	GB10249	4	1	28.5	LOC551947	Poly(ADP-ribose) glycohydrolase	8,551	
	GB17400	4	1	24.7	LOC726618	Similar to Alhambra CG1070-PD	4,400	
	GB10983	4	2	19.0	LOC411219	Metastasis suppressor protein 1	1,365	173 (egg)
	GB15909	4	1	12.0	ewg	DNA-binding protein Ewg	3,230	
	GB12244	4	3	8.9	LOC725706	rna-binding protein pno1-like	7,437	208 (egg)
Thelytokous embryos	GB15671		4	−61.7	LOC409309	E3 ubiquitin-protein ligase UBR4-like, purity of essence-like	1,728	221 (egg)
	GB14218		4	−57.7	LOC410796	PHD finger protein 2-like, JmjC domain-containing histone demethylation protein 1D/E/F	2,945	
	GB14495		4	−44.5	LOC408497	Uncharacterized LOC408497, SAP domain	1,413	
	GB10959		4	−36.8	LOC727029	26S proteasome non-ATPase regulatory subunit 1-like	1,007	
	GB10818		4	−25.4	LOC100577325	Uncharacterized LOC100577325	51

### Bisulfite PCR validation of candidate DMGs

**Fig. 6:**
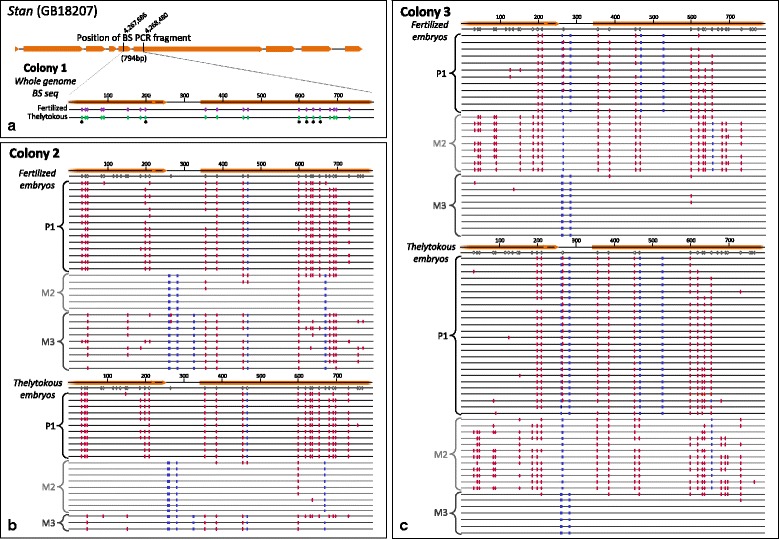
Direct sequencing of bisulfite PCR products of *Stan* (GB18207) from colony 2 and 3 fertilized and thelytokous embryos. **a** Summary of the 794 bp region of *Stan* used for bisulfite PCR sequencing. The location of *Stan* exons are indicated in orange. Methylated sites determined in the whole genome bisulfite sequencing of colony 1 are shown for fertilized (*purple*) and thelytokous (*green*). The * indicates CG sites that were deemed as hypermethylated in fertilized embryos, where 6 of the 7 hypermethylated sites are encompassed by the bisulfite PCR region. **b**&**c** Allele-specific methylation patterns of colony 2 (**b**) and colony 3 (**c**) fertilized and thelytokous embryos. The total number of CG sites present in the bisulfite PCR region is indicated as open ovals below exons (*orange*). Methylated CG sites are indicated as red ovals, and SNPs are designated as blue rectangles. The 3 alleles are labeled P1 (allele of paternal origin) and M2 and M3 (alleles of maternal origin)

If genomic imprinting occurs in Capensis, we predict that genes inherited from males and females will be differentially methylated at some sites. If so, paternally methylated genes would be hypermethylated in fertilized (queen laid) embryos relative to unfertilized (worker-laid) embryos. Accordingly, we determined if patterns of methylation differed depending on whether they were inherited from a male or a female. When examining whole genome bisulfite sequencing data, it is difficult to trace methylation back to an original paternal or maternal allele due to the removal of many informative SNPs during conversion of unmethylated cytosines to thymine. The problem is exacerbated by short reads, which often uncouple any remaining informative SNPs from methylation haplotypes. Therefore, we used bisulfite PCR encompassing larger fragments of candidate genes to investigate allele-specific methylation. We selected three candidate genes that contained a number of hypermethylated sites in fertilized eggs, *Stan* (GB18207, Fig. 6), *Stoned B* (GB17165, Additional file 1: Figure S2) and *Sap30* (GB18386, Additional file 1: Figure S3) to examine the level of methylation in the fertilized and thelytokous embryos collected from our two remaining colonies, 2 and 3. To determine the parent of origin of each allele, we sequenced the same gene in the fathering male. Thus for fertilized eggs we could identify which allele was paternal and which was maternal. In thelytokous eggs we could determine whether an allele was originally derived from the grandmother or the grandfather.

Sequences of *Stan* showed three distinct alleles in each colony, via informative G-to-A SNPs. Within a colony, fertilized and thelytokous embryos carried the same three alleles (Colony 2, Fig. 6b; Colony 3, Fig. 6c). Based on sequences of *Stan* obtained from the drone fathers used as sperm donors in colonies 2 and 3, we assigned the paternally-derived allele as allele P1, and inferred the maternally derived queen alleles as allele M2 and M3 for each colony (Fig. 6b & c).

The methylation patterns of each *Stan* allele varied considerably. In Colony 2, allele P1 showed the highest level of methylation, with an average of 51 % of the 31 possible CG sites methylated in fertilized embryos and 56 % in thelytokous embryos (Fig. 6b, Table 4). Allele M2 showed the lowest levels of methylation, with 10 % (fertilized) and 3 % (thelytokous), and M3 was intermediate with 34 % (fertilized) and 28 % (thelytokous). In Colony 3, P1 had intermediate methylation levels (fertilized 28 %, thelytokous 32 %), M2 the highest (fertilized 61 %, thelytokous 51 %) and M3 the lowest (fertilized 2 %, thelytokous 5 %; Table 4). Thus while the percentage of methylation and the location of methylated sites between alleles differed greatly, within-allele methylation was similar between fertilized and thelytokous samples in the two colonies (Fig. 6b&c).

**Table 4:** Percent methylation of the three alleles present in colony 2 and colony 3 for the *Stan* (GB18702) gene

		% Methylation (at 31 CG sites)		
	Allele origin	Fertilized	Thelytokous	Methylation level
AI2				
Allele 1 (P1)	*Paternal*	51	56	*High*
Allele 2 (M2)	*Maternal*	10	3	*Low*
Allele 3 (M3)	*Maternal*	34	28	*Intermediate*
AI3				
Allele 1 (P1)	*Paternal*	28	32	*Intermediate*
Allele 2 (M2)	*Maternal*	61	51	*High*
Allele 3 (M3)	*Maternal*	2	5	*Low*

The degree of methylation in *Stoned B* in Colony 2 and Colony 3 was much lower than the methylation present in the whole genome bisulfite sequence of Colony 1 fertilized and thelytokous eggs, and not all of the three alleles could be identified based on SNPs. Thus a comparison of allele-specific methylation could not be made for this gene, although there was some evidence for differential methylation in amplicons where two alleles could be distinguished (Additional file 1: Figure S2).

Due to lack of informative SNPs, we could not distinguish between the three alleles in *Sap30*, although for this gene methylation levels were similarly high to the whole genome bisulfite sequence in colony 1 (Additional file 1: Figure S3). In colony 2, three of the 32 CG sites present were significantly more highly methylated in fertilized embryos and one more highly methylated in thelytokous embryos; and in colony 3, two CG sites were significantly more highly methylated in fertilized embryos (Additional file 1: Figure S3).

## Discussion

If imprinting occurs in honey bees, we would expect to see a difference in the methylation patterns of bi-parental, fertilized embryos produced by union of an egg and sperm, compared to uni-parental, thelytokous embryos produced by parthenogenesis. Here we have shown that embryos derived from the union of a paternal and maternal genome had about 10 % more total methylated CG sites (Table 1), twice as many hypermethylated CG sites (Fig. 4) and twice as many hypermethylated genes (Fig. 2b) than genetically identical embryos that arose from the union of two maternal genomes. Candidate genes that are parentally-imprinted may therefore exist among the hypermethylated genes. This finding enhances earlier theoretical [18–20], phenotypic [34–36], and genomic [33] evidence that honey bees use their DNA methylation system to imprint certain genes in a parent-of-origin specific manner.

Biologically and technically replicated bisulfite sequencing of one of the top hypermethylated genes in fertilized eggs, *Stan,* revealed allele-specific methylation. The three alleles in each of colonies 2 and 3 had differing methylation patterns that were strongly correlated with genotype. This allele-specific methylation was the same between fertilized and theytokous embryos, and is therefore inconsistent with paternal or maternal imprinting: methylation was the same regardless of the sex of the parent of origin (Fig. 6).

While the most intensely studied examples of allele-specific methylation relate to parent-of-origin imprinting, it has been reported that the most widespread form of allele-specific DNA methylation in humans is determined by DNA sequence in *cis* [10, 66]. Sequence-dependent or *cis*-mediated allele-specific methylation has been mapped to the human genome [67] revealing many SNPs and genomic regions that are associated with variation in DNA methylation [68]. While allele-specific methylation resulting from genomic imprinting is a non-Mendelian process, *cis-*mediated allele-specific methylation that is genetically determined is inherited in a Mendelian manner. An epigenetic mechanism that is genetically determined creates another level of genome regulation where the genotype and epigenotype are both involved in generating the phenotype.

The mechanisms behind genetically-determined allele-specific methylation are unclear [66]. The allele-specific methylation observed in *Stan* included one G-to-A SNP that resulted in the removal of a CG site, thus physically interrupting the process of methylation. However the majority of methylation differences were not as clear and may be a result of long-range affects of polymorphisms that modify chromatin structure and affinity of DNA binding proteins [66].

Mono-alleleic methylation leading to allele-specific gene expression has previously been reported in ants [69] and bumblebees [70], and in both cases the authors hypothesised that differences were due to parent-of-origin effects. An alternate explanation is that the observed mono-alleleic methylation was due to *cis*-mediated allele-specific methylation, and that sequence-specific methylation is widespread in Hymenoptera. There is one other report of *cis*-mediated allele-specific methylation in honey bees [71], where differentially methylated obligatory epialleles of the *AmLAM* locus were correlated with sequence variation and resulted in different transcription levels. If allele-specific methylation is as widespread as it is in the human genome, it may have a major impact on the epigenetic contribution to phenotype in Hymenoptera.

Whole genome bisulfite sequence analysis indicated that *Stan* was hypermethylated in fertilized embryos compared to thelytokous embryos at multiple CG sites but bisulfite PCR validation in Colonies 2 and 3 did not support this conclusion. The difference in our genomic comparison may have arisen from unequal sampling of each of the three alleles present in Colony 1, such that more highly methylated alleles were over-represented in the genomic reads in our fertilized egg sample. In the whole genome bisulfite data, the mean coverage for *Stan* was 9.2 reads in fertilized embryos and 12 reads for thelytokous embryos. At this level of coverage, it is possible that the three alleles were unequally covered.

Whole genome bisulfite sequencing has previously been used to compare methylation patterns between social insect castes with contrasting results [44, 69, 72]. Allele specific methylation may confound genome-wide analysis of differential methylation in any species where there is *cis-*mediated allele-specific methylation. This kind of problem may be particularly acute in social insect species like honey bees where queens mate with multiple males. For example, if we compare groups of workers of different ages or tasks and conclude that their methylation patterns are different, our conclusion may be confounded by non-equal representation of alleles that have inherently different patterns of methylation. Recent evidence suggests that lack of biological replication could result in sample-specific, rather than caste-specific differences, leading to incorrect conclusions arising from genome-wide methylation patterns [72]. Where possible, an experimental design that enables biological replication or alternative methods of validation is required to support conclusions of differential methylation. A combination of high-coverage analysis [64], single drone inseminated queens [71] and reciprocal crosses [34, 37] should allow specific alleles to be followed and provide a means to definitively distinguish between parent-of-origin and *cis-*mediated allele-specific methylation.

## Conclusions

We have shown that global DNA methylation patterns differ between diploid embryos produced sexually and by thelytokous parthenogenesis. Our experiment minimized differences between embryos that were unrelated to uni- or bi-parental origin. These patterns of differential methylation between bi-parental and uni-parental eggs are consistent with imprinting. Nonetheless we caution that at least some of these patterns may be a consequence of *cis*-mediated allele specific methylation. In particular, our demonstration of allele-specific methylation in *Stan* means that not all epigenetic differences between between bi-parental and uni-parental embryos can be attributed to parent of origin effects.

### Supporting data

Whole genome bisulfite sequencing data generated in this study have been deposited in Genbank BioProject under the following accession numbers: (http://www.ncbi.nlm.nih.gov/bioproject/PRJNA282063).

### Ethics statement

Studies were conducted using *Apis mellifera capensis,* an invertebrate animal that is neither endangered or protected and does not require ethics approval under the *Animal Research Act 1985,* NSW Government, Australia. The apiaries for sample collection were maintained at the ARC-Plant Protection Research Institute, Stellenbosch, South Africa. Samples were preserved in ethanol and imported under quarantine import permit IP14019459 to our quarantine approved facility (QAP #N2083) at the University of Sydney, Australia where they were processed in accordance with quarantine procedures.

## Additional files

Additional file 1:
**Figure S1.** Comparison of fold coverage at methylated CG sites in thelytokous and fertilized embryos. Both samples exhibit a median coverage of 5 reads, thus our comparison of differential methylation was restricted to CG sites with at least 5 reads in both samples. **Figure S2.** Direct sequencing of bisulfite PCR products of *Stoned* *B* (GB17165) from colony 2 and 3 fertilized and thelytokous embryos. Red ovals indicated methylated cytosines, blue squares indicate SNPs. **Figure S3.** Direct sequencing of bisulfite PCR products of *Sap30* (GB18386) from colony 2 and 3 fertilized and thelytokous embryos. Red ovals indicate methylated cytosines, open circles indicate total number of CG sites in the region, and dashed lines indicate incomplete sequence reads. Also shown is the methylation patterns in fertilized and thelytokous embryos from whole genome bisulfite sequencing of Colony 1. Asterisk indicates CG sites that are hypermethylated in fertilized embryos, dagger indicates CG sites hypermethylated in thelyokous embryos. **Table S1.** Non-CG methylation in Fertilised and Thelytokous embryos and comparison to *Apis mellifera* Capensis SNPs. **Table S2.** Primers used in bisulfite nested PCRs of *Stan* (Fig. 6), *Stoned B* (Additional file 1: Figure S2), *Sap30* (Additional file 1: Figure S3, *Syd* and *Pcl*. (PPTX 607 kb) Additional file 2: Genes with hypermethylated sites (25 % > methylation difference) in Fertilized embryos. Genes with hypermethylated sites (25 % > methylation difference) in Thelytokous embryos. Genes with a combination of hypermethylated sites (25 % > methylation difference) from both Fertilized and Thelytokous embryos. (XLSX 60 kb)

## Abbreviations

Capensis: *Apis mellifera capensis*
DMG: Differentially methylated gene
SNP: Single nucleotide polymorphism
