# Supplementary material for: Parent-of-origin effects on genome-wide DNA methylation in the Cape honey bee (Apis mellifera capensis) may be confounded by allele-specific methylation
Source: BMC Genomics. 2016 Mar 12;17:226. doi: 10.1186/s12864-016-2506-8 (PMC4788913; doi:10.1186/s12864-016-2506-8)
Supplement: Additional file 1: — Figure S1. Comparison of fold coverage at methylated CG sites in thelytokous and fertilized embryos. Both samples exhibit a median coverage of 5 reads, thus our comparison of differential methylation was restricted to CG sites with at least 5 reads in both samples. Figure S2. Direct sequencing of bisulfite PCR products of Stoned B (GB17165) from colony 2 and 3 fertilized and thelytokous embryos. Red ovals indicated methylated cytosines, blue squares indicate SNPs. Figure S3. Direct sequencing of bisulfite PCR products of Sap30 (GB18386) from colony 2 and 3 fertilized and thelytokous embryos. Red ovals indicate methylated cytosines, open circles indicate total number of CG sites in the region, and dashed lines indicate incomplete sequence reads. Also shown is the methylation patterns in fertilized and thelytokous embryos from whole genome bisulfite sequencing of Colony 1. Asterisk indicates CG sites that are hypermethylated in fertilized embryos, dagger indicates CG sites hypermethylated in thelyokous embryos. Table S1. Non-CG methylation in Fertilised and Thelytokous embryos and comparison to Apis mellifera Capensis SNPs. Table S2. Primers used in bisulfite nested PCRs of Stan (Fig. 6), Stoned B (Additional file 1: Figure S2), Sap30 (Additional file 1: Figure S3, Syd and Pcl. (PPTX 607 kb) [file 12864_2016_2506_MOESM1_ESM.pptx]

## Slide 1
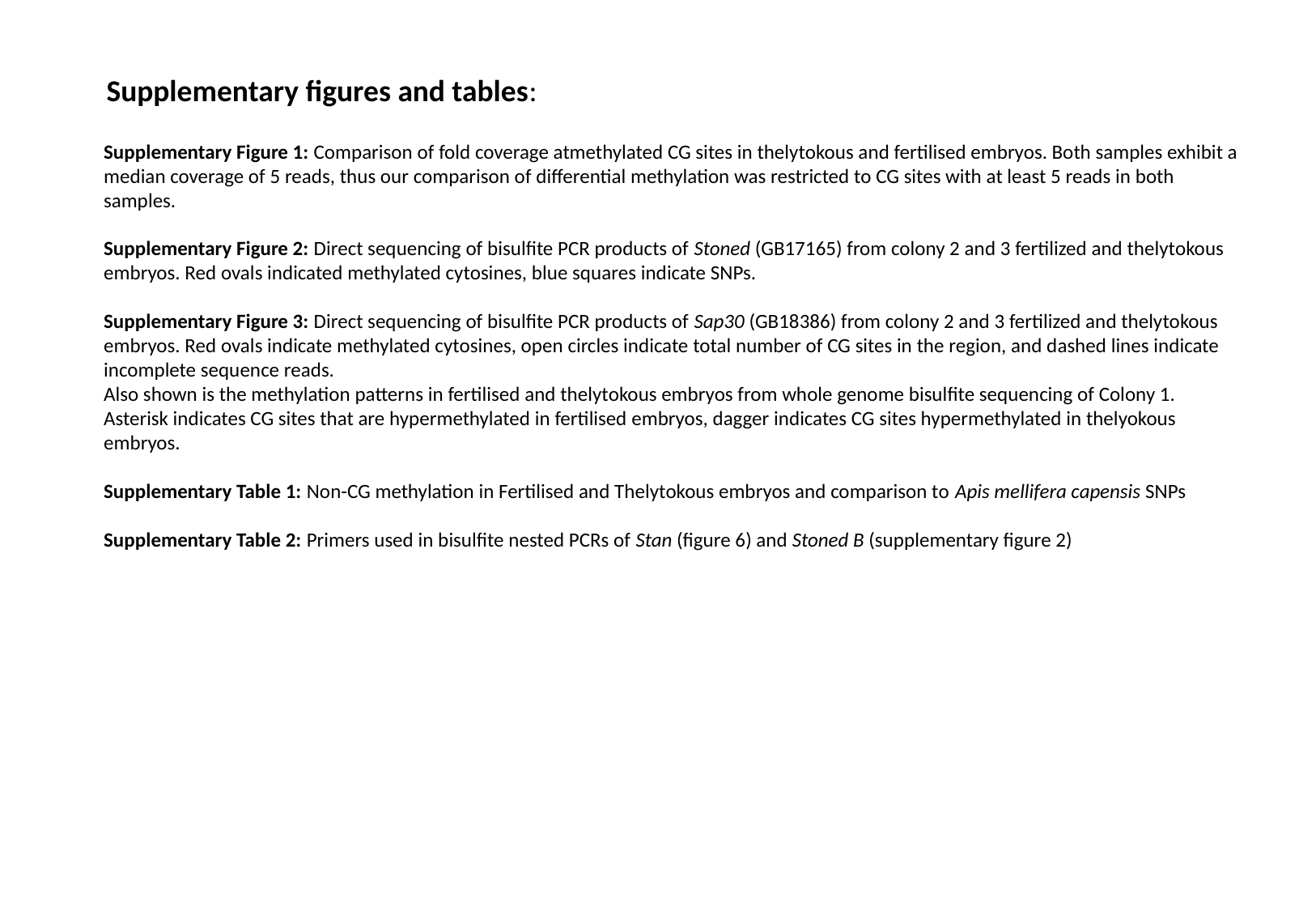

Supplementary figures and tables:
Supplementary Figure 1: Comparison of fold coverage atmethylated CG sites in thelytokous and fertilised embryos. Both samples exhibit a median coverage of 5 reads, thus our comparison of differential methylation was restricted to CG sites with at least 5 reads in both samples.
Supplementary Figure 2: Direct sequencing of bisulfite PCR products of Stoned (GB17165) from colony 2 and 3 fertilized and thelytokous embryos. Red ovals indicated methylated cytosines, blue squares indicate SNPs.
Supplementary Figure 3: Direct sequencing of bisulfite PCR products of Sap30 (GB18386) from colony 2 and 3 fertilized and thelytokous embryos. Red ovals indicate methylated cytosines, open circles indicate total number of CG sites in the region, and dashed lines indicate incomplete sequence reads.
Also shown is the methylation patterns in fertilised and thelytokous embryos from whole genome bisulfite sequencing of Colony 1. Asterisk indicates CG sites that are hypermethylated in fertilised embryos, dagger indicates CG sites hypermethylated in thelyokous embryos.
Supplementary Table 1: Non-CG methylation in Fertilised and Thelytokous embryos and comparison to Apis mellifera capensis SNPs
Supplementary Table 2: Primers used in bisulfite nested PCRs of Stan (figure 6) and Stoned B (supplementary figure 2)

## Slide 2
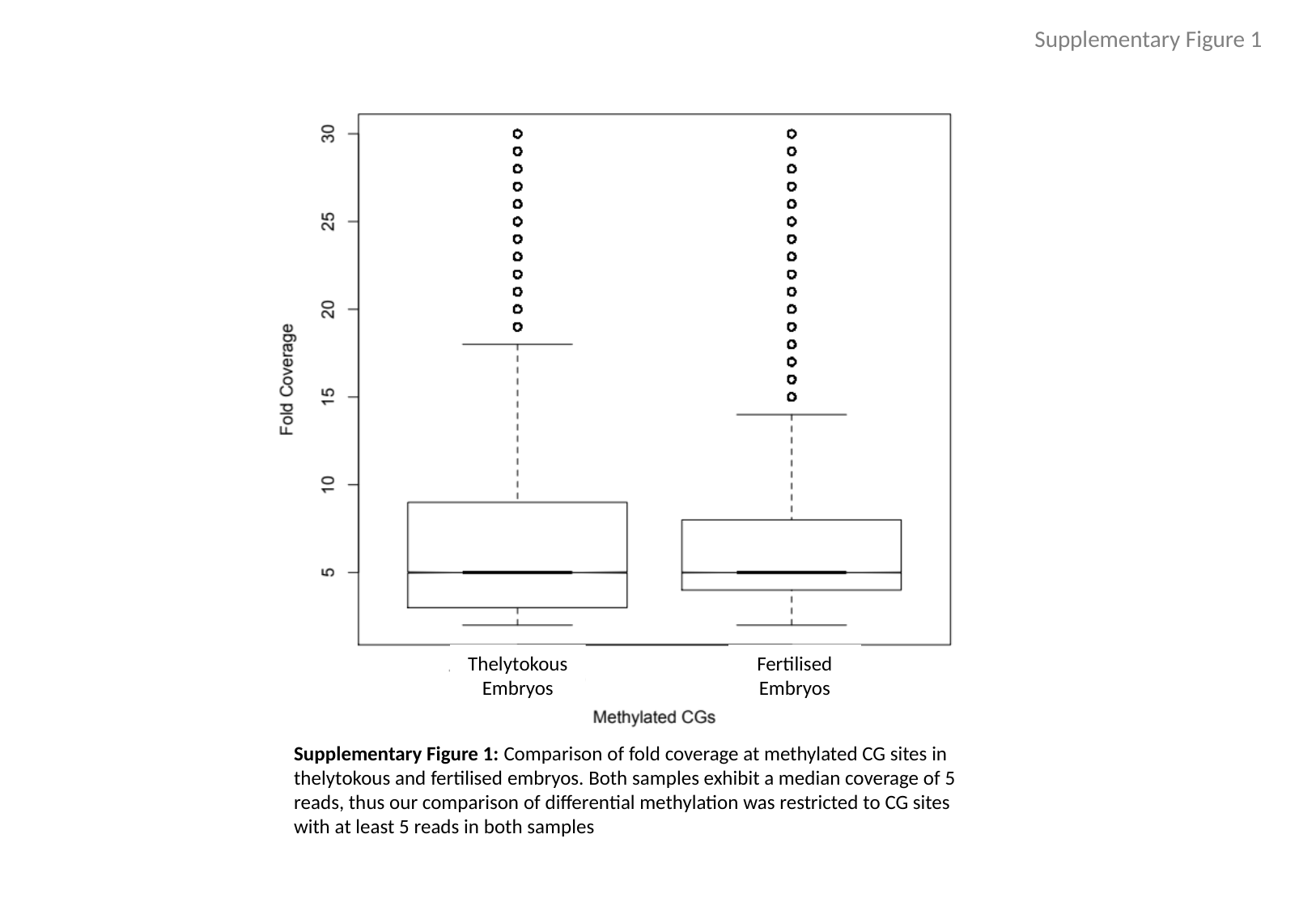

Supplementary Figure 1
Thelytokous
Embryos
Fertilised
Embryos
Supplementary Figure 1: Comparison of fold coverage at methylated CG sites in thelytokous and fertilised embryos. Both samples exhibit a median coverage of 5 reads, thus our comparison of differential methylation was restricted to CG sites with at least 5 reads in both samples

## Slide 3
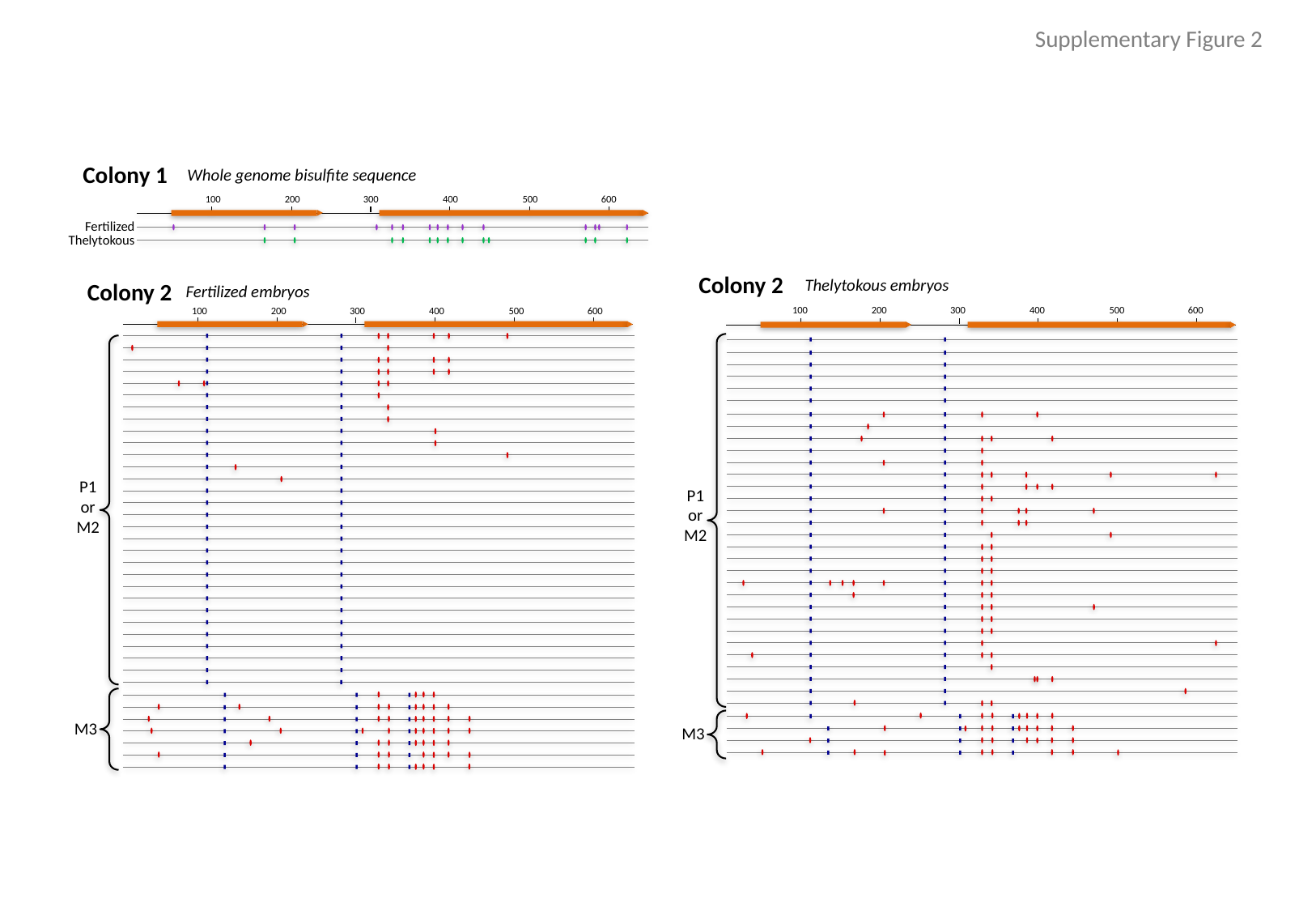

Supplementary Figure 2
Colony 1
100
200
300
400
500
600
Fertilized
Thelytokous
Whole genome bisulfite sequence
Colony 2
Thelytokous embryos
100
200
300
400
500
600
P1
or
M2
M3
Colony 2
Fertilized embryos
100
200
300
400
500
600
P1
or
M2
M3

## Slide 4
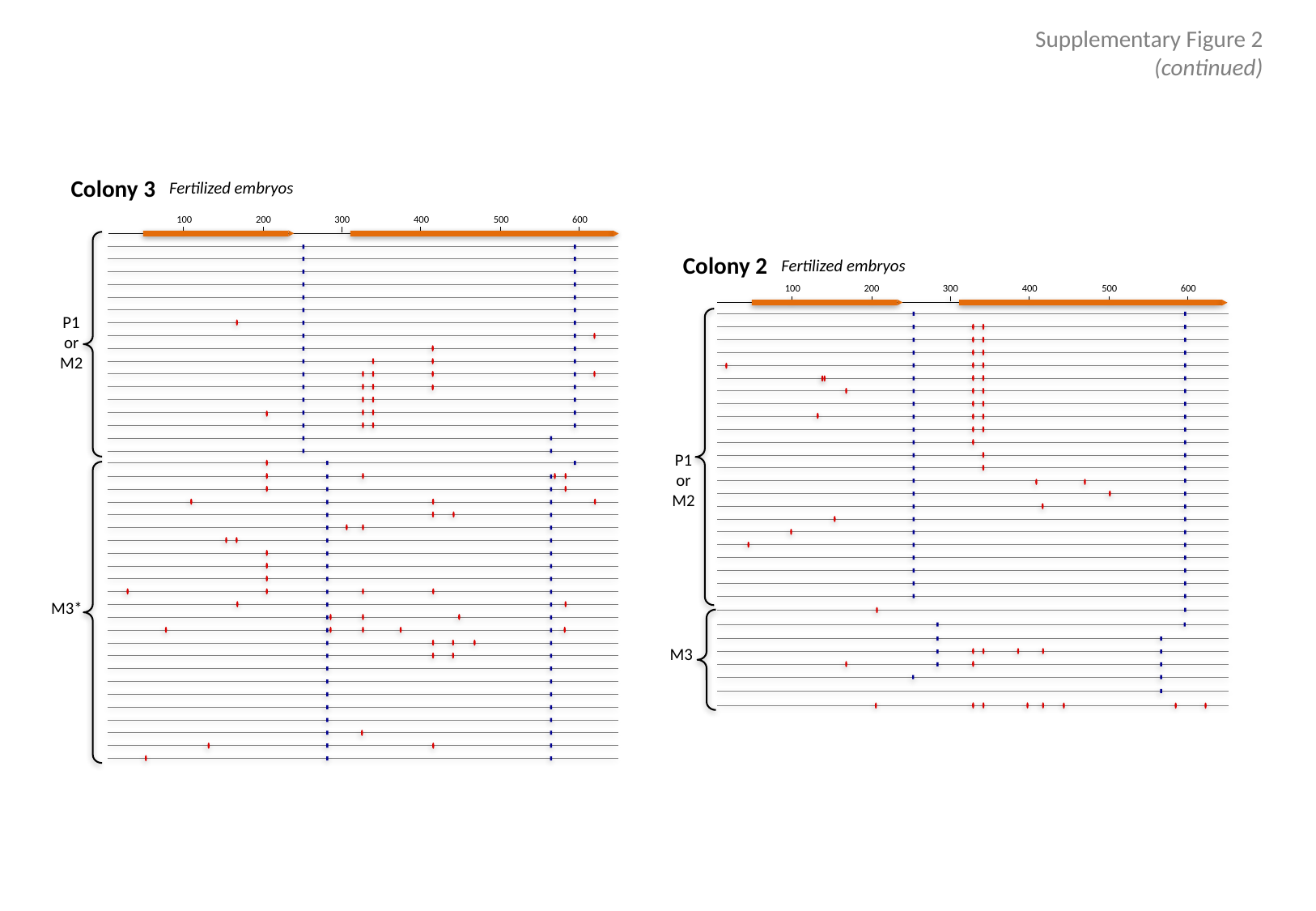

Supplementary Figure 2
(continued)
Colony 3
Fertilized embryos
100
200
300
400
500
600
Colony 2
Fertilized embryos
100
200
300
400
500
600
P1
or
M2
P1
or
M2
M3*
M3

## Slide 5
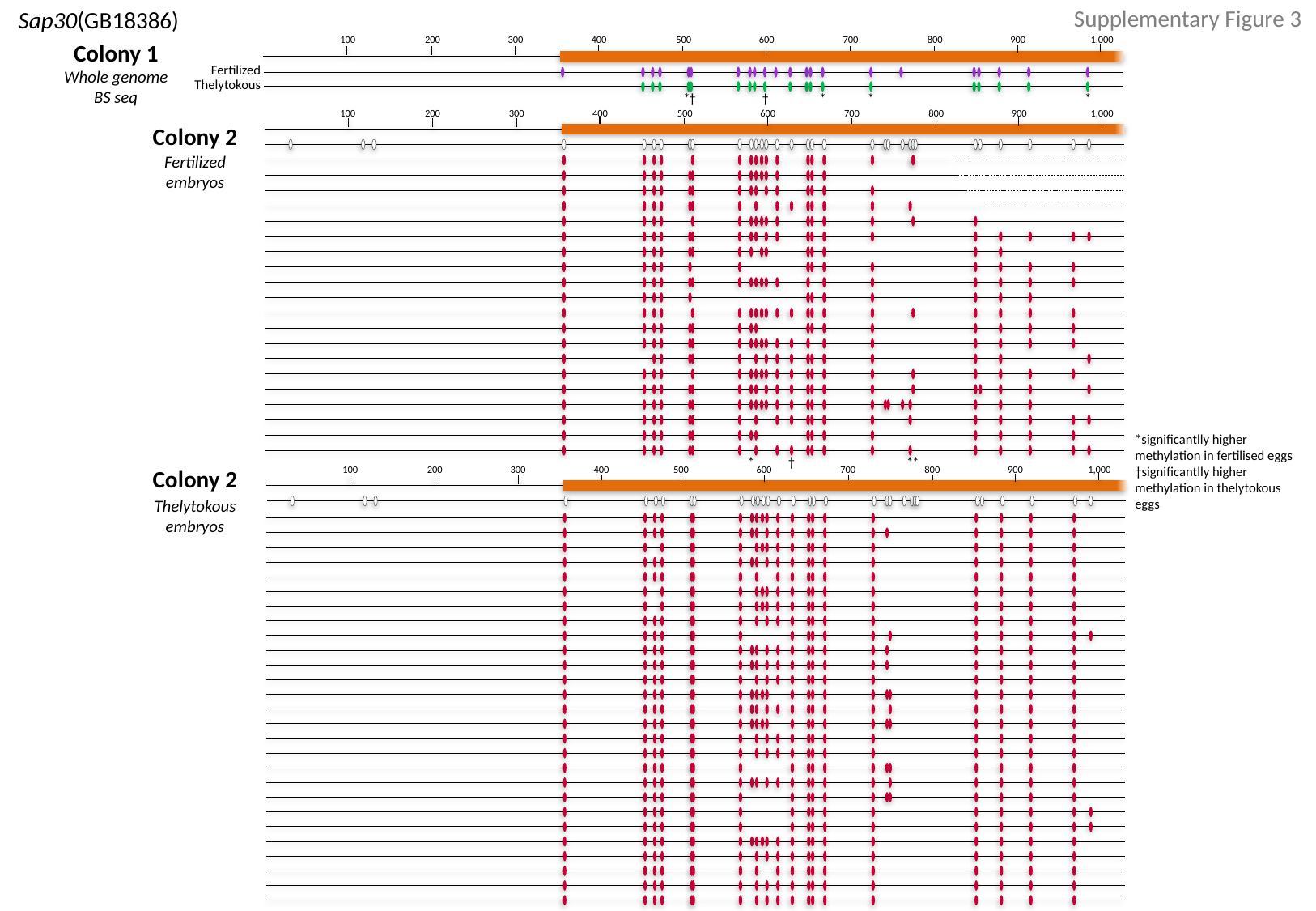

Supplementary Figure 3
Sap30(GB18386)
100
200
300
400
500
600
700
800
900
1,000
Fertilized
Thelytokous
*
†
†
*
*
*
Colony 1
Whole genome
BS seq
100
200
300
400
500
600
700
800
900
1,000
Colony 2
Fertilized
embryos
*significantlly higher methylation in fertilised eggs
†significantlly higher methylation in thelytokous eggs
†
*
*
*
100
200
300
400
500
600
700
800
900
1,000
Colony 2
Thelytokous
embryos

## Slide 6
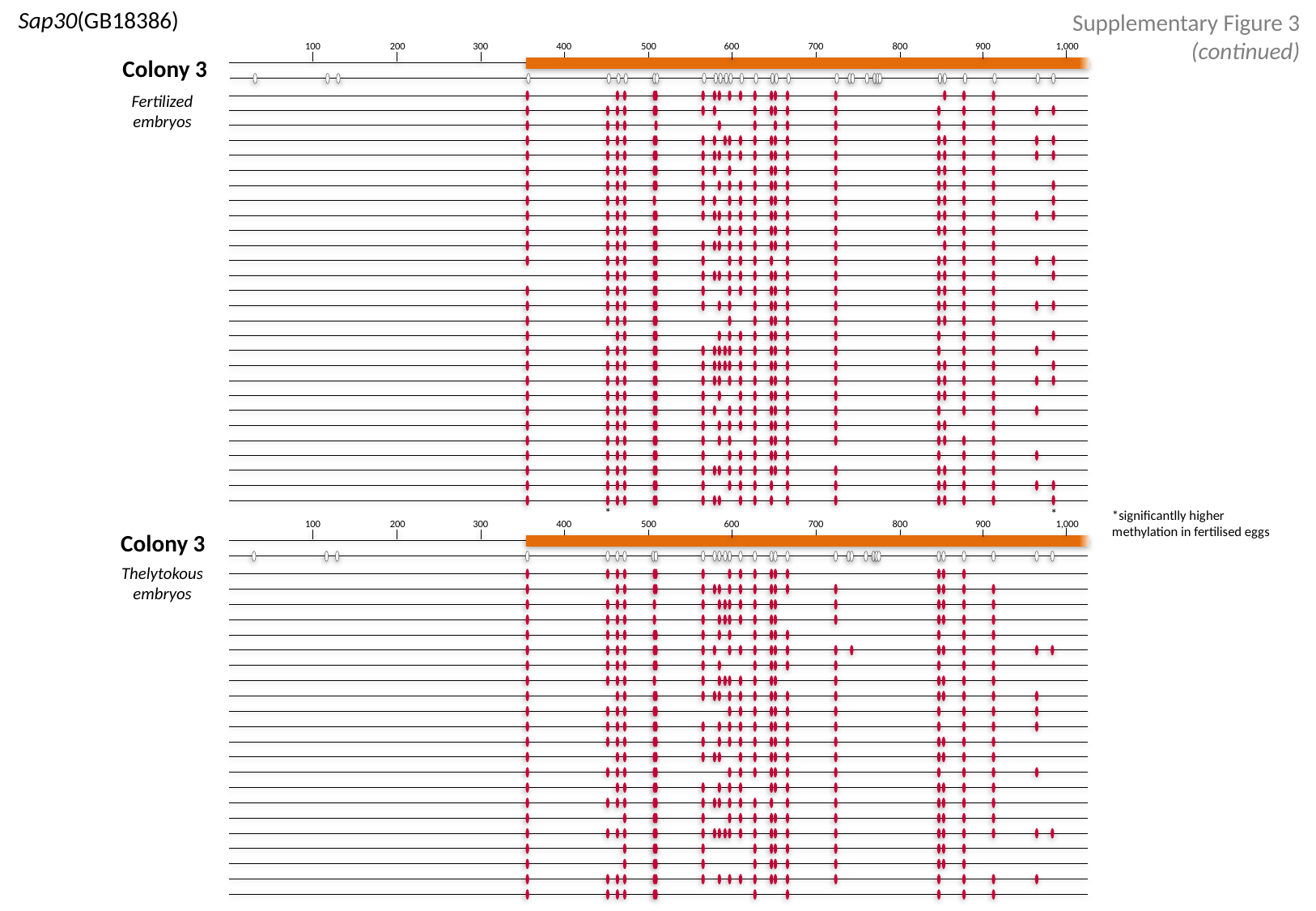

Sap30(GB18386)
Supplementary Figure 3
(continued)
100
200
300
400
500
600
700
800
900
1,000
Colony 3
Fertilized
embryos
*
*
*significantlly higher methylation in fertilised eggs
100
200
300
400
500
600
700
800
900
1,000
Colony 3
Thelytokous
embryos

## Slide 7
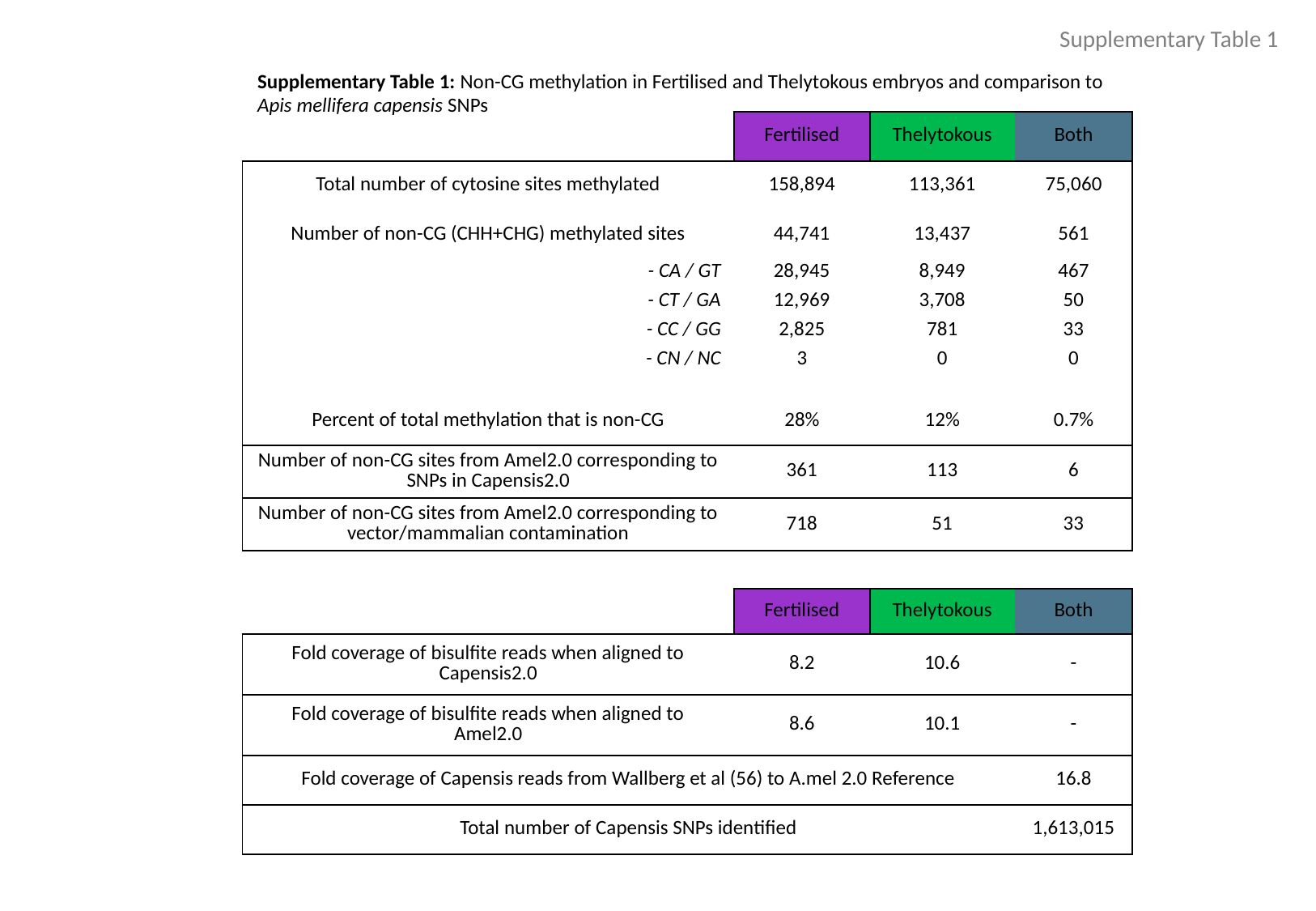

Supplementary Table 1
Supplementary Table 1: Non-CG methylation in Fertilised and Thelytokous embryos and comparison to Apis mellifera capensis SNPs
| | Fertilised | Thelytokous | Both |
| --- | --- | --- | --- |
| Total number of cytosine sites methylated | 158,894 | 113,361 | 75,060 |
| Number of non-CG (CHH+CHG) methylated sites | 44,741 | 13,437 | 561 |
| - CA / GT | 28,945 | 8,949 | 467 |
| - CT / GA | 12,969 | 3,708 | 50 |
| - CC / GG | 2,825 | 781 | 33 |
| - CN / NC | 3 | 0 | 0 |
| Percent of total methylation that is non-CG | 28% | 12% | 0.7% |
| Number of non-CG sites from Amel2.0 corresponding to SNPs in Capensis2.0 | 361 | 113 | 6 |
| Number of non-CG sites from Amel2.0 corresponding to vector/mammalian contamination | 718 | 51 | 33 |
| | Fertilised | Thelytokous | Both |
| --- | --- | --- | --- |
| Fold coverage of bisulfite reads when aligned to Capensis2.0 | 8.2 | 10.6 | - |
| Fold coverage of bisulfite reads when aligned to Amel2.0 | 8.6 | 10.1 | - |
| Fold coverage of Capensis reads from Wallberg et al (56) to A.mel 2.0 Reference | | | 16.8 |
| Total number of Capensis SNPs identified | | | 1,613,015 |

## Slide 8
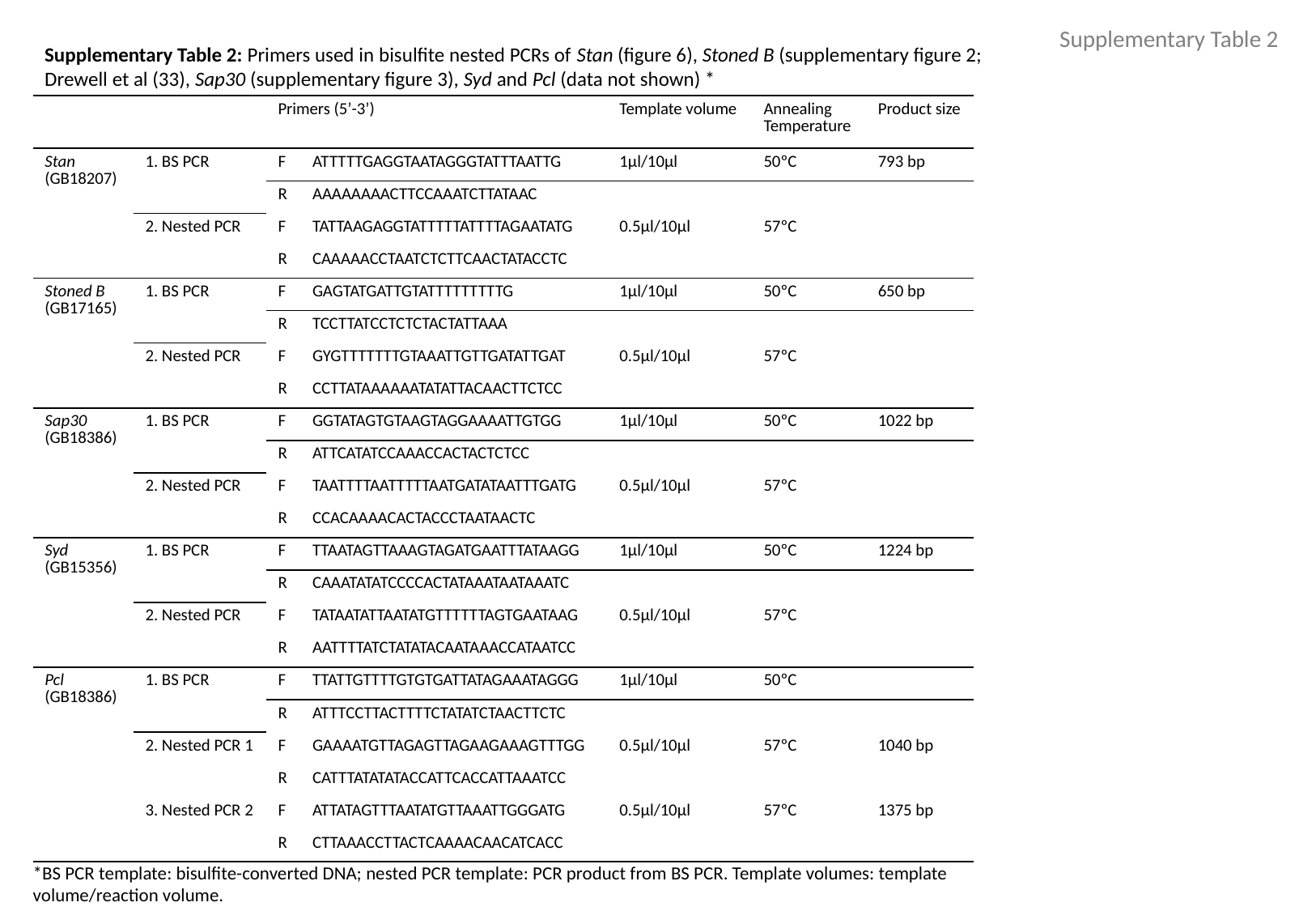

Supplementary Table 2
Supplementary Table 2: Primers used in bisulfite nested PCRs of Stan (figure 6), Stoned B (supplementary figure 2; Drewell et al (33), Sap30 (supplementary figure 3), Syd and Pcl (data not shown) *
| | | Primers (5’-3’) | | Template volume | Annealing Temperature | Product size |
| --- | --- | --- | --- | --- | --- | --- |
| Stan (GB18207) | 1. BS PCR | F | ATTTTTGAGGTAATAGGGTATTTAATTG | 1µl/10µl | 50ºC | 793 bp |
| | | R | AAAAAAAACTTCCAAATCTTATAAC | | | |
| | 2. Nested PCR | F | TATTAAGAGGTATTTTTATTTTAGAATATG | 0.5µl/10µl | 57ºC | |
| | | R | CAAAAACCTAATCTCTTCAACTATACCTC | | | |
| Stoned B (GB17165) | 1. BS PCR | F | GAGTATGATTGTATTTTTTTTTG | 1µl/10µl | 50ºC | 650 bp |
| | | R | TCCTTATCCTCTCTACTATTAAA | | | |
| | 2. Nested PCR | F | GYGTTTTTTTGTAAATTGTTGATATTGAT | 0.5µl/10µl | 57ºC | |
| | | R | CCTTATAAAAAATATATTACAACTTCTCC | | | |
| Sap30 (GB18386) | 1. BS PCR | F | GGTATAGTGTAAGTAGGAAAATTGTGG | 1µl/10µl | 50ºC | 1022 bp |
| | | R | ATTCATATCCAAACCACTACTCTCC | | | |
| | 2. Nested PCR | F | TAATTTTAATTTTTAATGATATAATTTGATG | 0.5µl/10µl | 57ºC | |
| | | R | CCACAAAACACTACCCTAATAACTC | | | |
| Syd (GB15356) | 1. BS PCR | F | TTAATAGTTAAAGTAGATGAATTTATAAGG | 1µl/10µl | 50ºC | 1224 bp |
| | | R | CAAATATATCCCCACTATAAATAATAAATC | | | |
| | 2. Nested PCR | F | TATAATATTAATATGTTTTTTAGTGAATAAG | 0.5µl/10µl | 57ºC | |
| | | R | AATTTTATCTATATACAATAAACCATAATCC | | | |
| Pcl (GB18386) | 1. BS PCR | F | TTATTGTTTTGTGTGATTATAGAAATAGGG | 1µl/10µl | 50ºC | |
| | | R | ATTTCCTTACTTTTCTATATCTAACTTCTC | | | |
| | 2. Nested PCR 1 | F | GAAAATGTTAGAGTTAGAAGAAAGTTTGG | 0.5µl/10µl | 57ºC | 1040 bp |
| | | R | CATTTATATATACCATTCACCATTAAATCC | | | |
| | 3. Nested PCR 2 | F | ATTATAGTTTAATATGTTAAATTGGGATG | 0.5µl/10µl | 57ºC | 1375 bp |
| | | R | CTTAAACCTTACTCAAAACAACATCACC | | | |
*BS PCR template: bisulfite-converted DNA; nested PCR template: PCR product from BS PCR. Template volumes: template volume/reaction volume.
